# Visual Outcomes of Pupilloplasty in Ocular Trauma and Iatrogenic Damage

**DOI:** 10.3390/jcm11113177

**Published:** 2022-06-02

**Authors:** Katarzyna Nowomiejska, Dariusz Haszcz, Katarzyna Adamczyk, Agnieszka Brzozowska, Vincenza Bonfiglio, Mario Damiano Toro, Robert Rejdak

**Affiliations:** 1 Chair and Department of General and Pediatric Ophthalmology, Medical University of Lublin, 20-079 Lublin, Poland; haszcz@poczta.onet.pl (D.H.); kadamczyk100@gmail.com (K.A.); toro.mario@email.it (M.D.T.); robertrejdak@yahoo.com (R.R.); 2 Department of Mathematics and Medical Biostatistics, Medical University of Lublin, 20-090 Lublin, Poland; agnieszka.brzozowska@umlub.pl; 3 Eye Clinic, Biomedicine, Neuroscience and Advance Diagnostic (BIND) Department, University of Palermo, 90143 Palermo, Italy; enzabonfiglio@gmail.com; 4 Eye Clinic, Public Health Department, University of Naples “Federico II”, 80131 Naples, Italy

**Keywords:** pupilloplasty, mydriasis, irydodialysis

## Abstract

Purpose: To report the visual outcomes of different techniques for iris pupilloplasty in eyes after traumatic and iatrogenic damage. Methods: 70 consecutive eyes with posttraumatic (80%) and postoperative (20%) iris damage were included. According to the preoperative diagnosis, the eyes were divided into three groups: mydriasis (50%), partial iris defects (24%), and iridodialysis (26%). Multiple techniques were performed: the Siepser slip-knot technique, the “lasso” technique, and suturing to the sclera. These techniques were combined in some cases. Results: The best improvement of visual acuity was found for the Siepser slip-knot technique with a median of 0.7 (SD ± 0.83) before surgery and 0.52 logMAR (SD ± 0.65) after surgery with regard to the surgical technique, and for mydriasis with a median of 0.7 (SD ± 0.75) before surgery and 0.52 logMAR (SD ± 0.49) after surgery with regard to preoperative diagnosis. Pupilloplasty was combined with additional surgery (corneal suturing, secondary intraocular lens implantation, anterior or pars plana vitrectomy) in 80% of cases. Apart from corneal suturing, all additional procedures ensured improvement in visual acuity. Conclusions: The slip-knot technique was the only suturing technique that resulted in a significant improvement in visual acuity. Other surgical procedures are usually needed in the majority of cases that undergo pupilloplasty, and they also give visual gain.

## 1. Introduction

Iris defects have been described in the literature for more than 100 years [1,2]. There are different types of iris damage. Traumatic iris damage includes sphincter tear, iris chaffing, tear at the root of the iris, iridodialysis, iris transillumination defects, and aniridia [3]. One type is the dilated atonic pupil, i.e., a slightly reactive pupil secondary to sphincter damage. Full or partial thickness defects can occur at the stromal level. The root of the iris can also be damaged, leading to iridodialysis. Iridodialysis is a complication resulting from trauma or the phacoemulsification procedure, since the iris root is the thinnest and weakest portion [4,5]. Other structures of the eye can be also damaged as a result of trauma, for example the cornea, lens, or posterior segment. Thus, additional surgery may be needed, including intraocular lens (IOL) scleral fixation [6] or pars plana vitrectomy [7]. 

In the past, iris prolapse was usually externalized and dissected [8,9]. Since the introduction of viscoelastics and modern instrumentation [10,11], iris surgery has enormously improved. Currently, there are several methods available for iris reconstruction, including iris implants as iris prosthesis [12] and suturing methods [13]. A number of prosthetic iris devices are available, including large-incision, rigid diaphragm [14], aniridic intraocular lens style devices, small-incision devices incorporating a capsular ring, and flexible, customized, small-incision iris prostheses [15]. Iris suturing methods aim to close the defect and/or reform the physiological pupil and include single or multiple interrupted iris sutures, running sutures using forceps for iris manipulation, and the single-pass four-throw technique [16,17,18]. Management options for iris defects are guided by the type of iris defect and the presence of other ocular comorbidities as traumatized eyes are very heterogenous. Pupilloplasty works best when the iris defect is small (<90°) and the remnant iris tissue is of sufficient quality and quantity to achieve a functional pupillary aperture [15]. The concept of minimally invasive pupilloplasty was first introduced by McCannel in 1976 [19]. Another technique is iris cerclage for the correction of persistent mydriasis due to diffuse iris sphincter dysfunction [20,21]. In the case of dialysis, suturing the iris to the sclera is necessary. Another option is sutureless cauterization of the iris stroma in the case of corectopia. Patients with partial or total iris defects suffer from functional disadvantages such as increased glare sensitivity, diplopia, photophobia, reduced visual acuity, and contrast sensitivity [22]. Thus, pupilloplasty should improve visual acuity, however the heterogeneity of eye trauma may reduce the effectiveness of the visual function results.

The purpose of this study was to report the visual outcomes of different iris suturing methods in patients with posttraumatic and postsurgical iris defects in a large eye trauma reference center.

## 2. Materials and Methods

The present study was designed as a retrospective observational case-control study conducted at the Chair and Department of General and Pediatric Ophthalmology of the Medical University of Lublin, Poland. The study was approved by the Ethics Committee of the Medical University of Lublin, Poland (approval number KE-0254/277/2021) and was performed in accordance with the tenets of the Declaration of Helsinki. Seventy consecutive patients with iris damage who were treated surgically between 2019 and 2021 were included in the study. The inclusion criteria were as follows: (1) iris injury due to trauma and (2) iris injury due to iatrogenic damage. The mean age was 51.60 ± 20.16 years (median 54.50 years). The proportion of males and females was 77% and 23%, respectively. 

Pre- and postoperative photographs were obtained with a digital camera (Topcon DC-4). All patients underwent a detailed ophthalmologic examination before and after surgery, including best-corrected visual acuity (BCVA) measured with projected light Snellen charts. The results of the visual acuity tests were converted into the logMAR (minimum angle of resolution) scale. Subjects with counting fingers, hand motion, light perception, or no light perception visual acuity were assigned logMAR values of 1.9, 2.3, 2.7, and 3.0, respectively [23]. Follow-up examinations were performed on the first day after surgery, after two weeks, and after three months. The mean follow-up duration was 4.3 ± 1.9 months. 

### 2.1. Surgical Technique

**Figure 1:**
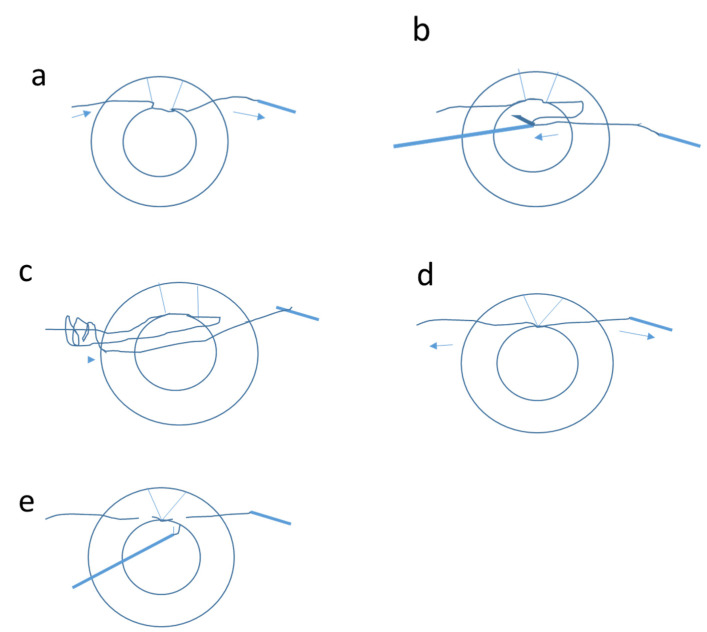
Schematic picture showing steps of Siepser slip-knot technique for iris suturing for focal defects: (**a**)—inserting the Prolene suture through the paracentesis; (**b**)—the peripheral loop of the suture is grasped with a Sinskey hook and brought through the opposite paracenthesis, bypassing the anterior chamber; (**c**)—a double-throw slipknot is then performed outside the anterior chamber; (**d**)—both sutures are extracted outwards, pulling the knots back into the anterior chamber and opposing the iris tissue at the edges; (**e**)—the suture ends are trimmed. Arrows indicate the direction of movement.

The surgeries were performed using local peribulbar anesthesia by one surgeon (D.H.). For the Siepser slip-knot technique, two paracenteses were made; next, a 10-0 Prolene suture was passed through the paracentesis (Figure 1a), and a large peripheral suture was left at either end. The peripheral loop was grasped with a Sinskey hook and brought through the opposite paracenthesis, bypassing the anterior chamber (Figure 1b). A double-throw slipknot was then performed (Figure 1c), and both sutures were extracted outwards, pulling the knots back into the anterior chamber and opposing the iris tissue at the edges (Figure 1d). Another knot was created similarly. Finally, the suture ends were trimmed (Figure 1e), and the same technique was repeated until the iris defect was closed [16].

**Figure 2:**
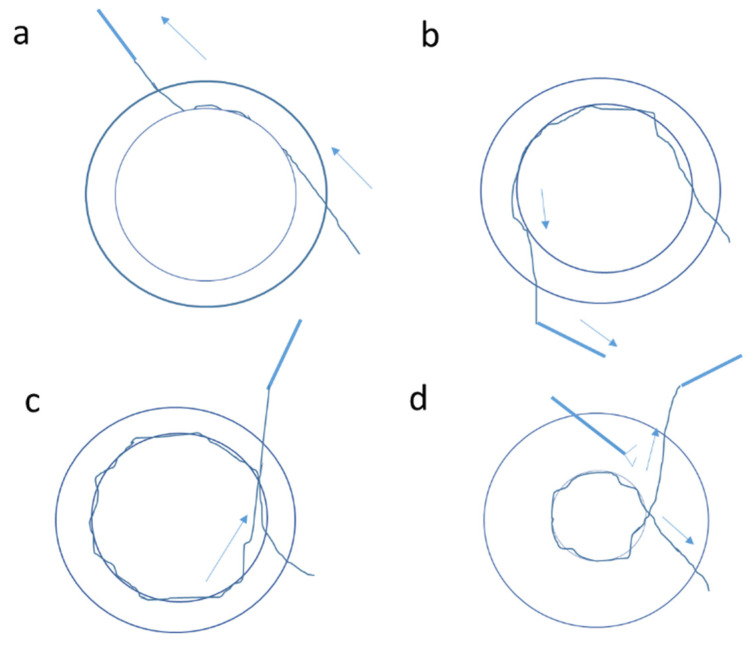
Schematic picture showing steps of the “lasso” technique for iris suturing for mydriasis: (**a**)—the first iris bite is taken at the peripheral pupillary edge at nine o’clock and this is repeated until a continuous row of three to four suture bites have been made in the inferior iris at five o’clock, (**b**)—the same loop is formed from one to five o’clock, (**c**)—it is continued from one to nine o’clock; (**d**)—the suture tension can be adjusted for the required pupillary size before applying the final knot and cutting the sutures. Arrows indicate the direction of movement.

For mydriasis, Siepser slip-knot technique sutures or the “lasso technique” were used. For this technique, a total of 3 limbal or clear corneal stab entries were fashioned at one, five, and nine o’clock. Viscoelastic was injected in the anterior chamber, and a 10-0 polypropylene suture was inserted through nine o’clock. Forceps were used to hold the iris tissue inserted from ten o’clock. The first iris bite was taken at the peripheral pupillary edge (Figure 2a), and this was repeated until a continuous row of three to four suture bites were made in the inferior iris at five o’clock. A loop was formed from one to five o’clock (Figure 2b), then one to nine o’clock (Figure 2c). Thus, three loops were formed at one, five, and nine o’clock. The suture tension was then adjusted for the required pupillary size before applying the final knot (Figure 2d) [24].

**Figure 3:**
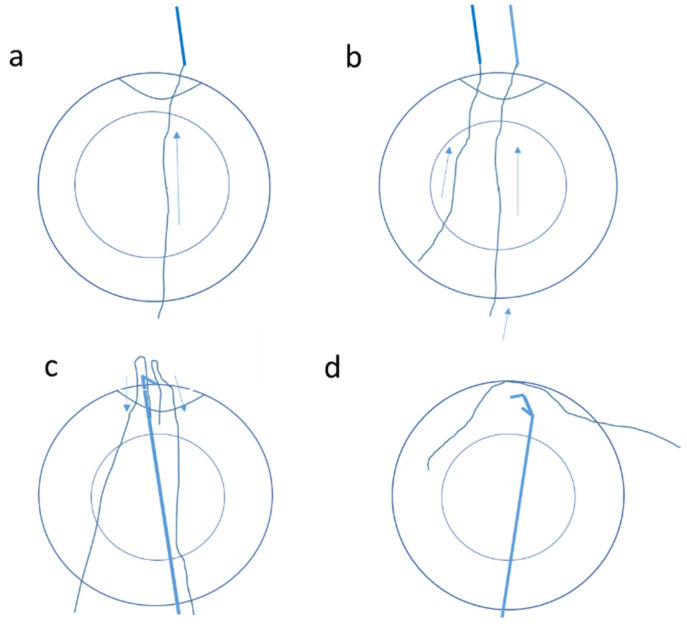
Schematic picture showing steps of the sclera suturing technique for iris suturing in the case of irydodialysis: (**a**)—the needle is passed from the torn iris and brought out from the sclera; (**b**)—the second needle is passed through the second paracenthesis out of the sclera; (**c**)—a Sinskey hook or iris hook can then bring the other end of both sutures to the anterior chamber; (**d**)—the suture ends are adequately tightened, the knots are buried, and the flaps are closed. Arrows indicate the direction of movement.

In the case of iridodialysis, suturing to the sclera was performed. In this technique, a 17 mm straight needle with a 10-0 polypropylene suture was used. A paracentesis was made 180 degrees away from the iridodialysis site. Along with the suture, the needle was passed through the torn iris and brought out from the sclera near the area of normal iris insertion (Figure 3). Similarly, a second paracentesis was fashioned and the second suture was passed out of the sclera (Figure 3b). A Sinskey hook or an iris hook then brought the other end of both sutures to the anterior chamber (Figure 3c). The suture ends were adequately tightened, the knots were buried, and the flaps were closed (Figure 3d) [25].

Cauterization is a sutureless technique [26,27] that can be performed in addition to suturing techniques in order to recenter or enlarge a small pupil. Endodiathermy cautery is applied to the iris stroma to induce tissue contraction and to gently pull the pupil in the meridian the probe is applied. 

Sometimes more than one technique was used to suture the iris and to recenter the pupil due to the heterogeneity of the trauma.

### 2.2. Statistical Analysis

The values of the analyzed measurable parameters were presented as the mean and standard deviation (SD), and non-measurable parameters were presented as counts and percentages. The Mann-Whitney test was used to compare independent groups. The normal distribution of the measurable parameters was assessed using the Shapiro-Wilk test. The non-parametric statistical Wilcoxon signed-rank test was used to compare dependent variables of the two related samples. The Chi2 test was used to assess differences between the qualitative features. Spearman’s rank correlation coefficient was used to assess the relationship between the variables. A level of significance of *p* < 0.05 indicated the existence of statistically significant differences. All calculations were performed using STATISTICA 13.0 (StatSoft, Krakow, Poland) software.

## 3. Results

### 3.1. Iris Damage

**Figure 4:**
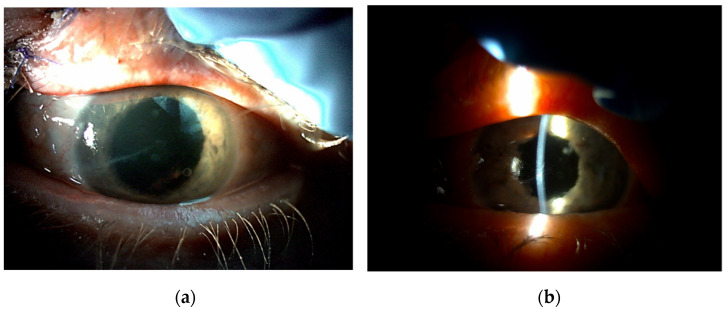
Posttraumatic mydriasis before (**a**) and after (**b**) pupilloplasty with “lasso” suture.

**Figure 5:**
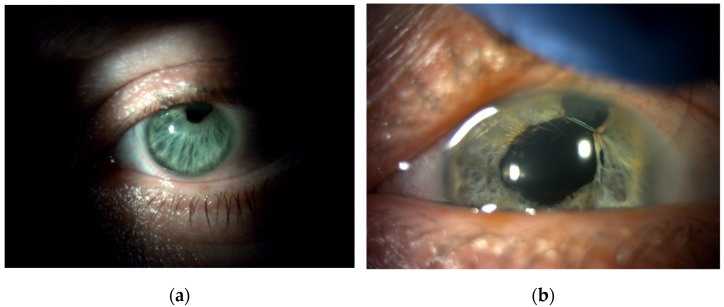
Focal postoperative iris defect before (**a**) and after (**b**) pupilloplasty with one suture on the sphincter and cauterization.

**Figure 6:**
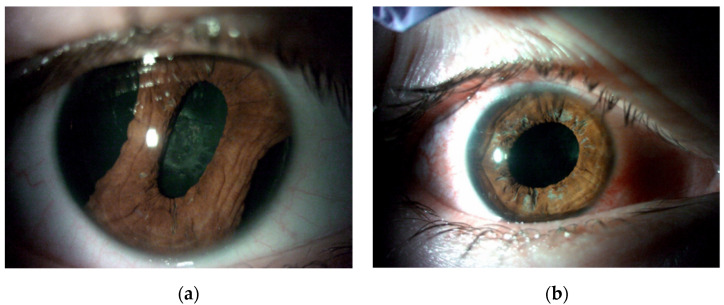
Posttraumatic iridodialysis before (**a**) and after (**b**) pupilloplasty with suturing to the sclera.

The patients were divided into three groups according to preoperative diagnosis (iris damage): mydriasis (*n* = 35; 50.00%) (Figure 4), partial iris defects (*n* = 17; 24.29%) (Figure 5), and iridodialysis (*n* = 18; 25.71%) (Figure 6). 

Trauma was the cause of iris damage in 80% (*n* = 56) of cases, while the remaining 20% (*n* = 14) were the result of postoperative damage (Table 1). 

### 3.2. Surgical Technique

Regarding the surgical technique, the most common surgical strategy was suture of the iris sphincter using the Siepser slip-knot technique (86%, *n* = 60), i.e., there was one suture in 44 of the cases (73%), two sutures in 11 of the cases (18%), and three sutures in 5 of the cases (8%). Suturing to the sclera was performed in eight eyes (11%): one suture was placed into the sclera in six eyes (75%), two sutures in one eye (12.5%), and three sutures in one eye (12.5%). A “lasso” suture was performed in four eyes (6%) (Table 2). Additional manipulations included synechiae removal (29%) and cauterization (11%).

One surgical technique was performed in 64.29% (*n* = 45) of patients, two combined techniques were performed in 24.29% (*n* = 17), and three techniques were performed in 11.42% (*n* = 8) of patients. (Supplementary Material—Combined slip-knot technique and suturing to the sclera in the case of irydodialysis).

No intraoperative complications were noted. The most prominent improvement to visual acuity was noted in the eyes operated with the Siepser slip-knot technique in both groups.

### 3.3. Visual Acuity

Overall, the visual acuity improved significantly (*p* = 0.000005) after surgery, with a median of 0.7 (SD ± 0.83) before surgery and 0.46 logMAR (SD ± 0.64) after surgery. 

Regarding the type of damage, the most pronounced improvement was achieved in the group that had a preoperative diagnosis of mydriasis (Table 3).

**Figure 7:**
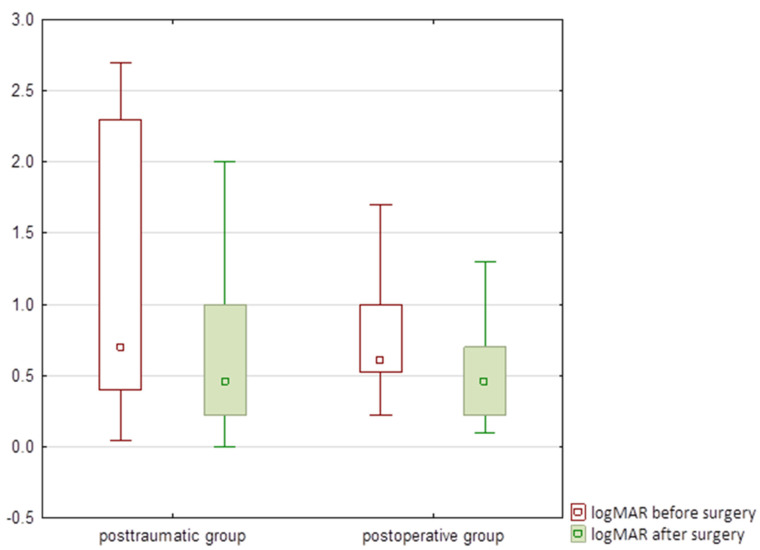
Comparison of pre- and postoperative logMAR visual acuity in the group with iris defects due to trauma (posttraumatic group) and due to intraoperative damage (postoperative group). Small box means median, large box means 25-75%, whisker means non-outlier range.

There were no significant differences (*p* = 0.75) in the pre- and postoperative visual acuity between the posttraumatic and postoperative cases, i.e., preoperative visual acuity was 0.7 logMAR in the posttraumatic group and 0.61 logMAR in the postoperative group, and postoperative visual acuity was 0.46 logMAR in both groups (Figure 7).

There was a significant improvement (*p* = 0.0004) in the visual acuity for the posttraumatic group, however there was no significant improvement for the postoperative group (*p* = 0.27).

There were no significant differences (*p* = 0.42) in the values of intraocular pressure before (median 16 mmHg) and after (15 mmHg) surgery. One case of secondary glaucoma was noted.

### 3.4. Additional Surgery

Pupilloplasty was performed as the only surgery in 14 cases (20%) and was combined with additional surgery in 56 cases (80%). The additional surgery was as follows: IOL reposition (*n* = 9; 13%), corneal suturing (*n* = 5; 7%), cataract removal with IOL implantation (*n* = 17; 24%), secondary IOL implantation (*n* = 41; 59%), anterior vitrectomy (*n* = 22; 31%), pars plana vitrectomy (*n* = 15; 21%), and Yamane scleral fixation (*n* = 26; 37%). The reasons for pars plana vitrectomy were retinal detachment (five cases), vitreous hemorrhage (eight cases), or dislocated lens (two cases). The visual acuity was significantly better in all groups, excluding patients subjected to corneal suturing in the posttraumatic group (Table 4). 

**Figure 8:**
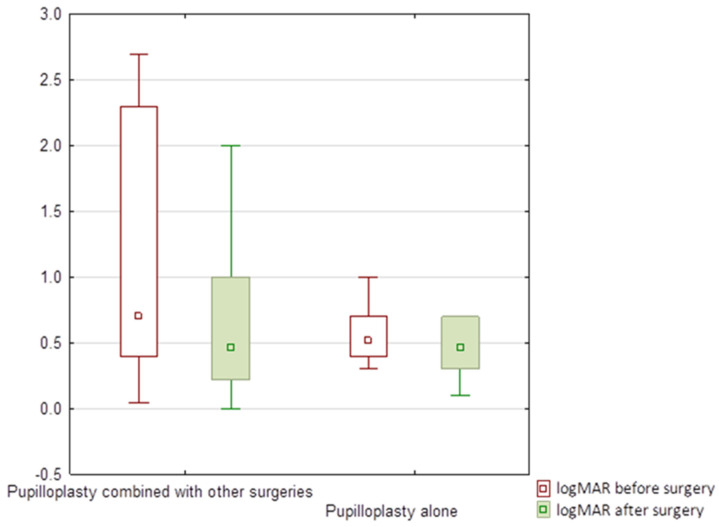
Log MAR visual acuity before and after pupilloplasty alone and pupilloplasty combined with other surgeries. Small box means median, large box means 25-75%, whisker means non-outlier range.

There were significant improvements in the logMAR visual acuity for eyes which underwent pupilloplasty alone and pupilloplasty combined with other surgical procedures that were mentioned earlier (Table 5 and Figure 8).

## 4. Discussion

The surgical reconstruction of eyes with iris defects is almost invariably complex and challenging as it requires precision and considerable surgical experience [15]. In the present study, we have analyzed the visual outcomes of pupilloplasty in posttraumatic and postoperative eyes. Different surgical strategies were used, depending on the nature of iris injury. To the best of our knowledge, this is the first study comparing the visual outcomes of different techniques of iris suturing in a large group of patients.

Visual acuity was significantly improved in 70 eyes that were treated surgically due to iris defects using different techniques in posttraumatic and postoperative eyes; visual improvement was the most pronounced in the group that was operated with the slip-knot technique. Regarding preoperative diagnosis, the best improvement was observed in patients with mydriasis.

Siepser’s sliding suture is conceptually more difficult to understand and more challenging to teach than other methods. However, the Siepser slip-knot technique is a very popular method for iris suturing as it maintains safety by using a small incision [28]. It has also been modified to stabilize the scleral fixation of IOL [29,30].

Improvements in visual function after pupilloplasty is unsurprising, as the iris helps to achieve good quality vision by reducing aberrations from the lens and cornea by decreasing the amount of light entering the pupil, preventing excessive glare, and enhancing the depth of focus [31]. An appropriate pupil diameter makes an additional pinhole effect and may contribute to better visual acuity. 

The majority of studies published so far in the field of pupilloplasty focus mainly on morphological outcomes, not visual outcomes [18], and refer to one surgical technique. The case series had limited numbers of patients, i.e., 27 eyes [18], 4 eyes [21], 12 eyes [32], or 8 eyes [30], or were just single case reports [33].

Traumatic and iatrogenic iris wounds do not heal on their own, and no fibrosis or scarring takes place. Thus, the aim of surgical treatment for iris defects is not only maintenance of the integrity of the anterior segment but also functional improvement and cosmetic concerns. Pupilloplasty can be effective for reapproximating the pupil shape and reducing the size of the iris defect, and therefore should be the first option considered in iris repair [15]. The limitations of pupilloplasty include the exacerbation of iris damage through intraoperative manipulation, excessive tension causing tears through sutures, poor approximation, insufficient reduction in pupil size, and unsatisfactory cosmetics [15]. 

Another option for posttraumatic iris defects is artificial silicone iris prosthesis (Humanoptics, Germany) [34]. This technique has very good cosmetic results as the iris is painted individually according to photographs of the contralateral iris. However, silicone iris prosthesis can cause a decrease or an increase in the intraocular pressure, corneal endothelial decompensation, or persisting inflammation. Moreover, the cost of this procedure exceeds other methods. Black diaphragm intraocular lenses (Morcher GmbH, Germany) [35] require large incisions of 10 mm, and the colors available are not customized. Glaucoma and corneal decompensation are complications that can affect postoperative visual acuity. These two methods are used in complete posttraumatic aniridia, after severe trauma, and also with other comorbidities. Suturing methods are more suitable for smaller iris defects or mydriasis.

Apart from iris damage, there are other changes in eyes as a consequence of trauma, e.g., aphakia, corneal scars, and retinal detachment, which make the surgery more complicated. In our study, the majority of eyes (80%) underwent additional surgical procedures, such as pars plana vitrectomy, anterior vitrectomy, corneal suturing, and primary or secondary IOL implantation. All procedures, apart from corneal suturing, led to improvements in visual acuity. 

The shortcomings of our study are that it is retrospective, and we did not use any imaging techniques to visualize the knot and iris position as this was done in a study by Kumar and colleagues [36]. They analyzed the results of spectral-domain optical coherence tomography in 26 patients and found that only one eye developed early knot loosening. This is important as we can presume that the normal iris ultrastructure can be altered after surgical procedures and intervening sutures. A significant change in iris configuration was noted after pupilloplasty, and a vertically oriented retained prolene suture was predominant with good endothelial vault.

The strengths of our study are that the number of patients is quite high (70 eyes), different techniques of iris suturing were performed, and both posttraumatic and postoperative cases were included.

## 5. Conclusions

In conclusion, pupilloplasty alone and when combined with other surgical procedures leads to improvements in visual acuity in eyes after ocular trauma and iatrogenic damage. The most prevalent and most effective technique regarding visual function was the Siepser slip-knot technique.

## Figures and Tables

**Table 1:** Causes of iris damage and types of iris damage in patients who underwent pupilloplasty.

Cause of Iris Damage	Type of Iris Damage			Total *n* (%)
	Mydriasis *n* (%)	Focal Defect *n* (%)	Dialysis *n* (%)	
Trauma	31	17	8	56
	55.36%	30.35%	14.29%	100.00%
Surgery	4	0	10	14
	28.57%	0.00%	71.43%	100.00%
Total	35	17	18	70
	50.00%	24.29%	25.71%	100.00%
Chi2 = 20.08; *p =* 0.00004 *				

* the results of statistical analysis.

**Table 2:** Improvement of the logMAR visual acuity depending on the surgical technique used for pupilloplasty. Asterisk means statistical significance (*p* < 0.05).

Type of Surgery	Time Span to Surgery	Mean	Median	Lower Quartile	Upper Quartile	Standard Deviation	Statistical Analysis	
							Z	*p*
Slip-knot technique *n* = 60	before	1.05	0.70	0.40	1.85	0.83	4.51	0.000007 *
	after	0.70	0.52	0.22	1.00	0.65		
Suture to the sclera *n* = 8	before	1.26	1.02	0.46	2.15	0.99	1.36	0.17
	after	0.76	0.26	0.13	1.50	0.91		
Lasso technique *n* = 4	before	0.44	0.45	0.23	0.65	0.26	0.00	1.00
	after	0.36	0.30	0.23	0.50	0.24		

**Table 3:** Improvement of LogMAR visual acuity with regard to preoperative diagnosis (type of damage). Asterisk means statistical significance (*p* < 0.05).

Type of Damage	Time Span to Surgery	Mean	Median	Lower Quartile	Upper Quartile	Standard Deviation	Statistical Analysis	
							Z	*p*
Mydriasis	before	0.87	0.70	0.40	1.00	0.75	3.02	0.003 *
	after	0.56	0.52	0.22	0.70	0.49		
Focal defect of the iris	before	1.22	0.70	0.40	2.30	0.98	2.31	0.02 *
	after	0.85	0.40	0.22	1.40	0.83		
Dialysis and synechiae	before	1.12	0.82	0.40	2.00	0.83	2.55	0.01 *
	after	0.76	0.55	0.22	1.00	0.70		

**Table 4:** LogMAR visual acuity before and after pupilloplasty, depending on the additional surgery. Asterisk means statistical significance (*p* < 0.05). IOL-intraocular lens.

Type of Surgery	Time Span to Surgery	Mean	Median	Lower Quartile	Upper Quartile	Standard Deviation	Statistical Analysis	
							Z	*p*
Corneal suturing	before	0.93	0.70	0.52	1.00	0.82	0.73	0.47
	after	0.79	0.70	0.15	1.40	0.75		
Cataract removal with IOL implantation	before	1.12	0.70	0.40	2.00	0.89	2.40	0.02 *
	after	0.56	0.22	0.15	1.00	0.68		
Secondary IOL implantation	before	1.17	0.82	0.40	2.30	0.90	3.81	0.0001 *
	after	0.67	0.40	0.22	1.00	0.61		
Anterior vitrectomy	before	1.08	0.70	0.40	2.00	0.84	3.10	0.002 *
	after	0.58	0.26	0.15	0.70	0.66		
Pars plana vitrectomy	before	1.70	2.30	0.70	2.30	0.90	2.38	0.02 *
	after	1.22	1.00	0.52	2.30	0.80		
Yamane scleral fixation	before	1.25	1.00	0.52	2.30	0.91	3.14	0.002 *
	after	0.76	0.56	0.30	1.00	0.60		

**Table 5:** LogMAR visual acuity before and after pupilloplasty alone and pupilloplasty combined with other surgeries. Asterisk means statistical significance (*p* < 0.05).

Type of Surgery	Time Span to Surgery	Mean	Median	Lower Quartile	Upper Quartile	Standard Deviation	Statistical Analysis	
							Z	*p*
Pupilloplasty alone	before	0.58	0.52	0.40	0.70	0.20	2.34	0.02 *
	after	0.45	0.46	0.30	0.70	0.20		
Pupilloplasty combined with other surgeries	before	1.13	0.70	0.40	2.30	0.90	4.02	0.0001 *
	after	0.74	0.46	0.22	1.00	0.70		

## Data Availability

Data are available upon request.

## Supplementary Materials

The following supporting information can be downloaded at: https://www.mdpi.com/article/10.3390/jcm11113177/s1, Video S1: Supplementary material—Combined slip-knot technique and suturing to the sclera in the case of irydodialysis.
